# Prognostic features of renal sarcomas (Review)

**DOI:** 10.3892/ol.2014.2838

**Published:** 2014-12-30

**Authors:** HAKAN ÖZTÜRK

**Affiliations:** Department of Urology, School of Medicine, Sifa University, Izmir 35240, Turkey

**Keywords:** renal sarcoma, sarcoma, prognosis

## Abstract

The aim of the present review was to evaluate the prognostic features of primary sarcomas of the kidney. A literature review was conducted using a number of databases, including Medline (PubMed) and Scopus, for studies published between January 1992 and December 2013. Of the studies published in English, those describing the prognostic features of primary sarcomas of the kidney were recorded. The electronic search was limited to the following keywords: Sarcoma, renal sarcoma, prognosis, diagnosis, immunohistochemistry, genetic and survey. Subsequent to the search, no review articles and/or meta-analyses associated with the prognosis of primary sarcomas of the kidney were identified. In total, 31 studies, which consisted of case studies, case series and studies concerned with the overall prognosis of urological soft-tissue sarcomas, were reviewed. Primary sarcoma of the kidney has a poor prognosis compared with other sarcomas of the urogenital system. In addition to the surgical excision of renal sarcomas, pathological, molecular and genetic prognostic factors are also considered. Due to the small number of cases, previous studies have not randomized the prognostic features of primary sarcomas of the kidney. The elucidation of the so-called ‘chaotic’ genetic and molecular basis of renal sarcomas will help to predict patient prognoses. Surgical excision is the most significant parameter for determining the prognosis of sarcomas of the kidney. However, sarcomas also exhibit prognostic features that are based upon pathological, genetic and molecular factors. The present review suggests that additional factors may be important in predicting the prognosis of patients with renal sarcomas, and that clinicians should plan treatment and follow-up regimens according to these factors.

## 1. Introduction

Overall, renal sarcomas account for 0.8–2.7% of malignant kidney tumors. Leiomyosarcomas (LMSs) are solitary lesions that are usually more common in females, and occur in the fourth and sixth decades of life. The primary symptoms of LMSs include pain, the presence of a palpable mass and hematuria. The tumors usually occur in the right kidney (1). LMSs account for 50–60% of kidney sarcomas; followed by liposarcomas in 10–15% of cases (2). Additional histological subtypes include osteogenic sarcoma, synovial sarcoma, rhabdomyosarcoma (RMS), fibrosarcoma, carcinosarcoma, malignant fibrous histiocytoma, angiosarcoma, anaplastic sarcoma, myeloid sarcoma, Ewing’s sarcoma/primitive neuroectodermal tumor (PNET), interdigitating dendritic cell sarcoma (IDCS) and malignant hemangiopericytoma (2). These tumors are avascular lesions, with the exception of hemangiosarcomas (2). Due to the lack of natural barriers for sarcomas arising from the mesenchymal components, renal sarcomas are able to expand and become large in size. Sarcomas typically possess a pseudocapsule, however, this represents an unreliable barrier and is often infiltrated by the tumor (3).

The ability to provide the prognosis of a cancer implies that the behavior of the tumor in terms of the diagnosis, treatment, recurrence, metastasis, response to therapy and survival is largely known. These data are invaluable for physicians and patients, and also with regard to public health. An accurate prognosis is critical in order to identify those individuals who are at risk, to predict survival rates, and to establish future strategies based upon the incidence and prevalence rates. The biological behaviors of renal and other soft-tissue sarcomas are unpredictable. However, the disease is known to be associated with a poor prognosis and a high metastatic potential (4). The lack of a suitable laboratory parameter, which would otherwise be used to monitor disease progression in renal sarcoma cases, represents another reason for continuous patient follow-up. Sarcomas develop by the malignant transformation of mesenchymal tissues, which are affected by mutagenic factors, and are associated with a poor prognosis. Compared with other types of urogenital sarcomas, such as those of the prostate and bladder, sarcomas of the kidney are less frequently observed (4). However, tumors of the kidney are associated with a poorer prognosis in terms of survival, and a lower life expectancy compared with other sarcomas of the urinary tract (4). The five-year survival rate is 82% in patients with retroperitoneal sarcoma, 73% in patients with sarcomas of the bladder, 44% in patients with prostate sarcoma and 39% in patients with sarcomas of the kidney (4).

Data from the American Joint Committee on Cancer (2010) revealed that the prognosis of soft-tissue sarcomas was directly associated with the disease stage (5). In addition to the tumor-node-metastasis (TNM) classification of renal sarcomas (Table I), the specific histological grade of the sarcoma is also used in tumor staging. However, the use of the TNM classification in the staging of sarcomas does not sufficiently predict the prognosis. Therefore, the histological grade is determined based upon the scoring system of the French Federation of Cancer Centers Sarcoma Group (Table II) (6).

Surgical excision and the stage of the tumor are the most important predictors of prognosis for cases of renal sarcoma. The present review evaluated other prognostic features for renal sarcoma cases.

## 2. Anatomical, surgical and radiological prognostic features

Surgical, anatomical and certain radiological features are important parameters for predicting the prognosis of patients with renal sarcomas. A review of the literature in the present study revealed that the most important prognostic factor for prognosis was surgical excision of the tumor. Valery *et al* (3) reported a survival rate of between 17.9 and 25 months in patients with LMS of the kidney. The prediction of prognosis is crucial in cases of renal sarcomas, which are characterized by a poor prognosis. In a study by Lewis *et al* (7), the presence of an unresectable tumor or an incomplete surgical resection were the factors most significantly associated with disease-specific mortality. The multivariate analyses from a further study by van Delan *et al*, which included 143 patients treated in the Netherlands, identified that complete tumor resection was associated with improved overall survival (8). The presence of reactive tissue surrounding the tumor is associated with a high risk of local recurrence. Therefore, resection of the tumor mass with large margins is often required. The removal of the reactive tissue, together with the tumor mass, is particularly recommended in cases where the reactive tissue surrounds the tumor. The most effective therapy for renal and retroperitoneal sarcomas is the removal and gross total resection of the tumor. In high-grade sarcoma cases, the toxic effects of radiotherapy on other organs limit the dose of radiotherapy, and therefore complicates control of the disease (9).

The presence of metastasis at the time of diagnosis is one of the most important predictors of prognosis, as the mean survival rate is usually shorter in those patients with metastatic disease at diagnosis. In a study by Dotan *et al* (10), the presence of metastasis at diagnosis, and a diagnosis of RMS, were identified as negative prognostic variables. In a study by Lee *et al* (4), the presence of metastasis at diagnosis was associated with a poor survival rate in univariate and multivariate analyses. Despite resection of the primary renal sarcoma, metastasis to the lungs, liver and colon is predictive of a poor prognosis (1). The presence of a lung metastasis originating from a renal sarcoma or the presence of a resectable lung metastasis has been identified to be associated with long-term disease-free survival in certain patients. However, the significance and contribution of other factors, such as the slow growth of the tumor, remain to be elucidated. The value of resecting liver metastases, in terms of survival, is also yet to be determined (11). In a study by Putnam and Roth (12), limited resection of lung metastases was suggested to be associated with long-term disease-free survival. However, the effect of a low tumor load, slow tumor growth and long disease-free intervals is yet to be elucidated.

An investigation of all findings reveals that surgical resection is the only prognostic factor able to confer increased survival rates for patients with a primary tumor, or for those who present with a primary tumor and metastatic disease. An inability to perform surgical resection appears to be the most unfavorable prognostic variable for overall survival (4). In terms of prognosis, there is no significant difference between males and females. Furthermore, the occurrence of the tumor on the right or left side of the body does not appear to affect prognosis. In addition, evaluation of the prognosis based upon age groups does not reveal any significant results (4). However, the prognosis is identified to be worse in symptomatic patients. A tumor size of <5 cm is a favorable prognostic feature (3). Kadikoy *et al* (13) evaluated the prognosis of patients with inoperable cases of bilateral renal sarcomas who had instead received neoadjuvant chemotherapy with gemcitabine and docetaxel, and revealed that bilateral tumor occurrence was predictive of a poor prognosis. Bilateral cancers may be affected by the same mutagenic factors, which suggests that tumor suppressor mechanisms may be dysfunctional.

Renal sarcomas commonly exhibit local recurrence. However, at present, there is no established chemotherapy protocol that can be followed in the case of local recurrence subsequent to docetaxel monotherapy or a combined treatment protocol. Therefore, disease recurrence, particularly following the administration of chemotherapy, is associated with a poor prognosis (14). On rare occasions, pre-operative chemotherapy or radiotherapy may aid in reducing the size of a previously unresectable tumor. The sensitivity of a tumor to chemotherapy and/or radiotherapy is regarded as a good prognostic feature (15). In a quantitative meta-analysis of 1,568 patients in 14 studies, doxorubicin-based adjuvant chemotherapy achieved a 6% disease-free survival rate (16). The studies, which included patients with renal sarcomas treated with post-operative chemotherapy protocols, evaluated treatment regimens, drug doses, sample size, tumor location and histological grades. Therefore, randomized controlled studies are required in order to evaluate survival benefits.

## 3. Pathological and immunohistochemical prognostic features

Compared with the subsequently listed cellular classification, a histological grade is able to describe the metastatic potential of a tumor with improved accuracy. A tumor grade is assigned according to the number of mitoses per high-power field, the degree of cellularity, the cellular and nuclear morphology, and the presence of necrosis. Discordance between pathologists with respect to tumor grading and histological subtype can be substantial (17). Dedifferentiated liposarcomas usually arise as a deep, atypical lipomatous tumor or as a well-differentiated liposarcoma with intermediate malignancy, due to a lack of metastatic capacity (5). Increased rates of necrosis, poor differentiation, mitotic activity and increased histological grade are associated with a poor prognosis. A study by Deyrup *et al* (18) revealed an association between the increasing histological grade of renal LMSs and the rate of survival. The histological grade was defined as a poor prognostic factor. Low-grade soft-tissue sarcomas exhibit limited metastatic potential, but also tend to recur locally. Therefore, surgical excision, which includes the removal of negative tissue margins measuring 1–2 cm or more in all directions, is the recommended treatment for patients with early-stage sarcomas (19).

The involvement of regional lymph nodes, although extremely rare in cases of kidney sarcoma, is predictive of a poorer prognosis. Certain subtypes of renal sarcomas are more likely to spread to the lymph nodes than others. These subtypes include high-grade RMS, vascular sarcoma, clear cell sarcoma and epithelioid sarcoma (20). Renal sarcomas that are sensitive to chemotherapy are associated with an improved prognosis. Cases of pediatric anaplastic kidney sarcoma have been reported. The most important feature of these tumors is the prospect that they may respond to chemotherapy (21). Myeloid sarcoma of the kidney often originates from leukemia cells, and has the potential to transform into acute myeloid leukemia. These types of sarcomas are also regarded as being sensitive to chemotherapy (22). In total, 60 cases of synovial sarcoma of the kidney, a subtype of renal sarcoma, have been reported in the literature. This histological subtype is associated with a poor prognosis. Synovial sarcoma is characterized by a specific t(X;18)(p11.2;q11.2) chromosomal translocation. The SS18-SSX fusion protein expressed during this translocation is believed to be associated with tumor pathogenesis (23). Synovial sarcomas exhibit a high potential for systemic metastasis. The tumor however is regarded to be sensitive to anthracycline-based chemotherapy, with response rates of up to 53%. The sensitivity of synovial sarcomas to chemotherapy-based therapies suggests the potential use of such regimens for young patients, or for patients hosting a large volume of tumor (24). Other tumor subtypes, such as Ewing’s sarcoma and PNET, are high-grade malignant tumors, which typically present in children and adolescents. The prognosis is worse in cases of metastatic disease. Ewing’s sarcoma and PNET of the kidney are rare, with just over 100 cases reported globally (25). PNET occurs in young adults, and is the predominant type of aggressive renal sarcoma in males aged 28–34 years old (26). Ewing’s sarcoma and PNET are considered to be systemic conditions that exhibit an aggressive course and are often characterized by early metastatic disease (25–50% at the time of presentation). Patients undergoing local therapy alone exhibit recurrence rates of 80–90%, which is likely to be due to the existence of subclinical metastasis at the time of the initial diagnosis (27). IDCS, a rare and only recently described subtype of renal sarcoma, originates from dendritic cells, a type of professional antigen-presenting cell that participates in the innate and adaptive immune response. IDCS is an extremely rare tumor that primarily occurs in the lymph nodes (28). The chromosomal translocation of the B-cell lymphoma 2 protein is believed to be associated with the pathogenesis of IDCS (29). Seven cases of embryonal RMS, another rare renal sarcoma, have been reported in the literature. High-grade RMSs are aggressive tumors with high metastatic potential and a tendency for lymph node metastasis. A total of four cases of undifferentiated sarcoma of the kidney have been previously reported. Poor differentiation is a negative prognostic feature for patients with renal sarcomas, as is the case for all other types of cancer (30).

There are contradictory reports regarding the value of histological subtypes in predicting prognosis. In a study by Lee *et al* (4), the histological subtype of the renal sarcoma was not defined as a prognostic factor for disease-specific survival in the univariate and multivariate analyses, as no association was observed between the histological subtype of the renal sarcoma and the rate of disease-specific survival. However, the small number of patients in the study complicated this statistical comparison.

## 4. Molecular and genetic prognostic features

Renal sarcomas are recognized as being genetically complex tumors, often bearing so-called ‘chaotic’ karyotypes, such as aneuploidy or polyploidy. However, studies are yet to report the existence of recurrent tumor-specific translocations (31). The studies in the literature have identified that the p16 and p53 tumor suppressor proteins are overexpressed in cases of LMS, and could therefore be used as prognostic markers in renal sarcomas (32). The mediator complex exhibits a key role in eukaryotic gene transcription activation. The mediator of RNA polymerase II transcription subunit 12 homolog (MED12) is a subunit of the mediator complex, which regulates the activity of the complex. A number of mutations at this location are believed to represent basic mechanisms involved in the development of sarcomas. The MED12 gene mutation occurs in uterine leiomyomas, but can also occur in pelvic and retroperitoneal LMSs. This suggests that different smooth muscle tumors develop as a result of similar mutagenic changes (33). The replication of DNA profiles obtained from cases of LMS revealed the presence of repetitive genomic changes. Of these genomic changes, a 17q duplication was revealed to be associated with long-term disease-free survival and a low risk of metastasis (3). In addition, 4q31 and 18q22 deletions were associated with a susceptibility for metastasis, and 1p33-p32.3 duplications were correlated with increased survival rates. The duplications at 1q21.3 were identified to be an independent prognostic marker for shorter survival times in patients with LMS, which suggested that genes located in this region may be involved in the aggressiveness of LMS (34). A further mutation believed to be involved in the pathogenesis of LMSs is the heterozygous mutation in the fumarate hydratase (FH) gene. Although this mutation was originally described for LMS of the skin, similar genetic mutations may be involved in the development of soft-tissue LMSs. The FH gene mutation exhibits familial inheritance, which may explain the occurrence of soft-tissue sarcomas at a young age in homozygous individuals. At present, limited data exists concerning the association between LMS and the FH gene mutation (35). The prune homolog 2 (PRUNE2) protein has an important role in cellular differentiation and the regulation of apoptosis. PRUNE2 demonstrates higher expression in small-sized tumors, particularly those measuring <10 mm. The expression of PRUNE2 has been identified to be downregulated according to increased tumor volume, with larger tumors associated with shorter survival times. In a study by Zhao *et al* (36), increased PRUNE2 protein expression was associated with a good prognosis in patients with LMS. The study revealed that increased PRUNE2 protein expression was an independent prognostic factor for overall survival in patients with LMSs. Furthermore, in a study by Tsiatis *et al* (37), c-Myc expression was reported to be a marker for poor prognosis in LMSs.

The anatomical, surgical, radiological, pathological, genetic and molecular prognostic features of renal sarcomas are summarized in Table III.

## 5. Conclusion

The prognosis of renal sarcomas, in light of the current literature, is difficult to predict. The most important positive prognostic feature for these tumors is complete surgical resection. Due to the presence of multiple histological subtypes of sarcomas, the specified prognostic features have not been evenly randomized. The sarcomas exhibit a ‘chaotic’ genetic and molecular structure. Previous studies, which have analyzed the overexpression of p16 and p53 genes, MED12 gene mutations, c-Myc expression, 4q31 and 18q22 deletions and FH mutations suggest that sarcomas possess a more complex molecular and genetic basis than is currently known or understood.

## Figures and Tables

**Table I:** Sarcoma stage according to the TNM system of the American Joint Committee on Cancer.

Stage	Tumor characteristics
IA	T1, N0, M0, G1 or GX: Tumor is ≤5 cm
IB	T2, N0, M0, G1 or GX: Tumor is >5 cm
IIA	T1, N0, M0, G2 or G3: Tumor is ≤5 cm
IIB	T2, N0, M0, G2: Tumor is >5 cm
III	T2, N0, M0, G3: Tumor is >5 cm; OR Any T, N1, M0 or G: Tumor can be any size
IV	Any T, N, M1 or G: Tumor can be any size

T, tumor; N, node; M, metastasis; G, grade; GX, the grade cannot be assessed.

**Table II:** Grading system based upon the French Federation of Cancer Centers Sarcoma Group.

Tumor differentiation	Mitotic count	Tumor necrosis	Histological grade
Score 1, good	Score 1, 0–9 mitoses per 10 HPF	Score 0, no necrosis	Grade 1; total score 2,3
Score 2, intermediate	Score 2, 10–19 mitoses per 10 HPF	Score 1, <50% tumor necrosis	Grade 2; total score 4,5
Score 3, poor	Score 3, ≥20 mitoses per 10 HPF	Score 2, ≥50% tumor necrosis	Grade 3; total score 6,7,8

HPF, high-power field.

**Table III:** Prognostic features of renal sarcomas.

Prognosis	Good prognostic features	Poor prognostic features
Surgical, anatomical and radiological	Complete surgical excision	Incomplete surgical excision
	Solitary tumor	Multiple or bilateral tumors
	Tumor diameter <5 cm	Tumor diameter >5 cm
	Negative lymph nodes	Positive lymph nodes
	Sensitivity to CTx and RTx	Presence of metastasis at the time of diagnosis
		Symptomatic patients
		Recurrence (particularly following surgery and CTx)
Pathological	Low grade	High grade
	Good differentiation	Poor differentiation
	Mitotic count <9 mitoses per 10 HPF	Mitotic count >20 mitoses per 10 HPF
	Tumor necrosis <50%	Tumor necrosis >50%
	Low Ki-67 proliferation index	A histological subtype of rhabdomyosarcoma, vascular sarcomas, clear cell sarcoma, epitheloid sarcoma, Ewing’s sarcoma or undifferentiated sarcoma
Molecular and genetic	17q duplications	Overexpression of p16 and p53
	1p33-p32.3 duplications	MED12 gene mutation
	PRUNE2 expression	4q31 and 18q22 deletions
		Fumarate hydratase mutation
		c-Myc expression

CTx, chemotherapy; RTx, radiotherapy; HPF, high-power field; PRUNE2, prune homolog 2.

## Abbreviations

LMS: leiomyosarcoma
RMS: rhabdomyosarcoma
PNET: primitive neuroectodermal tumor
IDCS: interdigitating dendritic cell sarcoma
TNM: tumor-node-metastasis
FH: fumarate hydratase
